# Suitability of the Kinect Sensor and Leap Motion Controller—A Literature Review

**DOI:** 10.3390/s19051072

**Published:** 2019-03-02

**Authors:** Tibor Guzsvinecz, Veronika Szucs, Cecilia Sik-Lanyi

**Affiliations:** Department of Electrical Engineering and Information Systems, University of Pannonia, 8200 Veszprem, Hungary; szucs@virt.uni-pannon.hu (V.S.); lanyi@almos.uni-pannon.hu (C.S.-L.)

**Keywords:** accuracy, gesture recognition, Kinect, human-computer interaction, human motion tracking, Leap Motion, precision, suitability

## Abstract

As the need for sensors increases with the inception of virtual reality, augmented reality and mixed reality, the purpose of this paper is to evaluate the suitability of the two Kinect devices and the Leap Motion Controller. When evaluating the suitability, the authors’ focus was on the state of the art, device comparison, accuracy, precision, existing gesture recognition algorithms and on the price of the devices. The aim of this study is to give an insight whether these devices could substitute more expensive sensors in the industry or on the market. While in general the answer is yes, it is not as easy as it seems: There are significant differences between the devices, even between the two Kinects, such as different measurement ranges, error distributions on each axis and changing depth precision relative to distance.

## 1. Introduction

**Figure 1:**
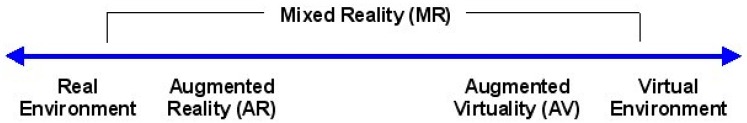
The types of realities, from real to virtual [4].

As technology progresses, the popularity of virtual reality, augmented reality and mixed reality increases. The uses of virtual reality [1], augmented reality [2] and mixed reality [3] are spread across multiple fields: education, military, medical fields, entertainment, etc. Each of the three mentioned realities have different locations on a “reality-scale”, which can be seen in Figure 1, and they also have different properties: virtual reality may be the simplest to use as it is a fully virtual, synthetic environment which can be interacted with. Augmented reality is different, because it is a reality which expands upon our reality. Mixed reality is between virtual and augmented reality and it allows augmented objects to be interacted with.

However, these realities need sensors to work as intended, meaning to place the user in a certain reality, so to say. To do this, the most common method is for the user to wear a Head-Mounted Display (HMD). Most HMDs have inertial sensors such as gyroscope and accelerometer to track the motion of the user’s head [5]. To fully realize the potential of these realities, tracking only the head is not enough as interaction with the environment is required with the use of other sensors, working together with the mentioned HMD.

**Figure 2:**
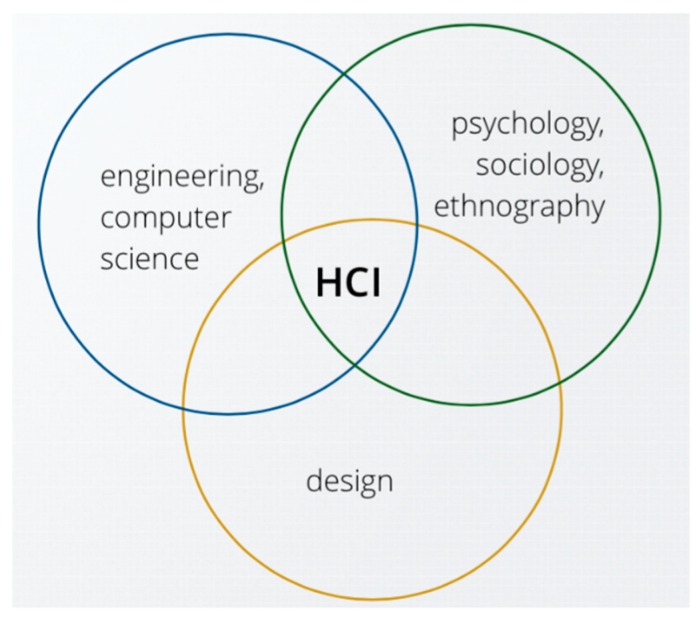
Parts of Human-Computer Interaction [7].

Additionally, the human—also known as the user – is an important element in the whole system which can be seen in Figure 2. Between the human and the machine, the interaction, usability and comfort are key factors. Human-Computer Interaction (HCI) is a multidisciplinary field of research which deals with these types of questions, not only on the hardware side but on the software side as well [6]. However, the engineering and the computer science sides of HCI are focused on in this paper, namely on the sensors in order to determine if they are viable as human motion tracking devices.

The problem with precise human motion tracking is that it is expensive and most people—especially those who live in developing countries—cannot afford it. Even if they could, most motion tracking sensors are not available commercially, possibly due to their use and price. A few of those sensors will be mentioned in this paper.

This paper investigates the use of low-cost sensors, such as the Kinect sensor (Microsoft, Redmond, WA, USA)—for full body motion tracking via its depth cameras and skeleton stream [8]—and the Leap Motion Controller (LMC, Leap Motion, San Francisco, CA, USA)—for hand motion tracking [9]—to see if they can substitute the expensive ones. The question of the Kinects and the LMC is interesting. This means that if they are applied in many fields due to being part of HCI studies, it should be determined if they are considered adequate as sensors by studying their precision and accuracy. Thus, the research question (RQ) of the authors is the following: Can these three sensors substitute expensive sensors while taking into account their accuracy, precision and price while also assessing existing gesture recognition algorithms?

The paper is structured as follows: Section 2 deals with the methodology used while reviewing and Section 3 briefly presents the devices and comments on their market situation. In Section 4, the state of the art is reviewed: the authors of this paper present existing uses of the two mentioned sensors. In doing so, the authors establish the suitability of the devices, meaning whether the devices are acceptable for certain tasks. In Section 5, the Kinect and the LMC are compared to similar price range sensors and to even more expensive ones based on their use, functionality and suitability. Section 5 also deals with the use of all sensors in existing applications. In Section 6, their accuracy and precision are examined. Existing algorithms are also presented and their accuracy are assessed. Finally, a proper conclusion is given in Section 7.

## 2. Methods

For this literature review the Web of Science, PubMed, and IEEE Xplore databases were searched using the following keywords for the Kinect devices: “Kinect” AND “review” OR “accuracy” OR “medical applications” OR “physical disability” OR “education” OR “gesture recognition” OR “precision” OR “skeleton”. For the LMC the following keywords were used: “Leap Motion” AND “review” OR “accuracy” OR “medical applications” OR “physical disability” OR “education” OR “gesture recognition” OR “precision”. The keywords “review”, “accuracy”, “precision”, “skeleton”, “gesture recognition” were selected because they offer the possibility of retrieving depth data or measurement information. The keywords also have the possibility of retrieving information about both accuracy and precision, even when talking about the skeleton stream or when recognizing gestures. While not synonyms, if a study focuses on precision, the accuracy is also always present, but the reverse is not the case when searching for keywords with “accuracy” in them while using at least one of the sensor names. In our case, when talking about measurement systems, accuracy means the degree of closeness of a measured quantity to its true value. Precision means the degree to which repeated measurements produce the same results under similar, unchanged conditions [10]. The keywords “medical applications”, “physical disability”, “education” were chosen to evaluate the multidisciplinary use of the sensors. Still, a low possibility exists that they present and evaluate algorithms that were developed by their authors. These algorithms use at least one of the sensors and usually, their accuracy is studied as well. Regarding the search, no filtering was applied: all articles were indexed since the inception of the first version of the Kinect [11] and the LMC [12]. Every author searched in one database. Both the search and the first part of the review were conducted from late-October 2018 to early–January 2019 and the second part in February 2019. The search results are presented in Table 1.

**Figure 3:**
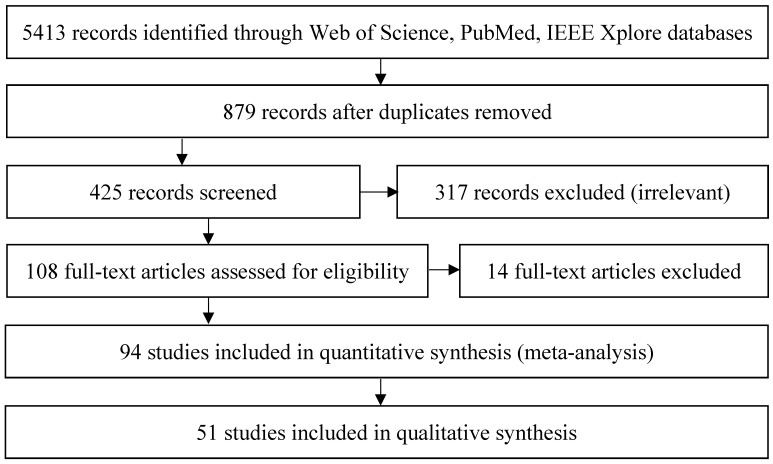
Selection of the relevant papers based on the Prisma 2009 Flow Diagram.

According to the search results the Kinect devices are more popular than the LMC and most of the studies regarding all the devices revolve around gesture recognition and their depth sensing properties. The number of studies regarding the precision of the devices is adequate, though the Kinects are more popular in this regard. However, there is a large overlap with accuracy and gesture recognition. After searching the databases, the selection of relevant studies was done according to the Prisma 2009 Flow Diagram [13] (see Figure 3 for details). After removing the duplicates, 425 records were screened. All three authors were part of the screening stage. The first two screening criteria were the titles and abstracts of the records. Three hundred and seventeen (317) records were excluded as neither their titles nor their abstracts referred to the state of the art, motion tracking, gesture recognition, the accuracy or precision of the devices. After that, 108 full-text articles assessed for eligibility which consisted of a content analysis. Based on their content, 14 full-text articles were excluded as those articles did not give significant information about the state of the art, motion tracking, gesture recognition, accuracy or precision. Out of these 14 articles, four were shorter versions of extended papers. Naturally, the shorter versions were omitted from the literature review. When doing the content analysis, each author analyzed different aspects.

The remaining 94 studies helped constitute the bulk of this research paper as those studies talked about the usefulness and suitability of the devices in multidisciplinary applications. Out of these, 51 studies helped with answering the authors’ RQ. The content of those papers is about human motion tracking while assessing the precision and accuracy of the devices or the accuracy of algorithms. 

## 3. Presenting the Sensors

Before talking about the state of the art, the devices should shortly be presented: The first Kinect was announced in 2009 [11] and the LMC in 2012 [12]. Both devices are popular with the public:

- Eight million Kinect units were sold in 60 days. In early-2013, the number of Kinects sold was over 24 million. During their lifetime, the almost 30 million Kinects units were sold, with 23 million being the Kinect v1 and the remaining being the Kinect v2 [14,15].
- In contrast, the LMC sold over 500 000 units after its first year on the market [16]. Since then, no new market data on the number of sold units was published. Additionally, Apple tried to acquire Leap Motion in 2018, but the deal was never finalized [17].

Sadly, Microsoft discontinued the Kinect sensors in the fall of 2017, and on 01.02.2018 the USB adapter for the Kinect devices was discontinued as well [15]. Without the adapter, the sensors could not connect to a PC. One of the reasons behind this discontinuation was that there were not enough good games for the Kinects. However, even if the entertainment industry was not satisfied with the Kinects, it is a different story in the field of research. Section 2 provides proof of this. In contrast to the Kinects, the LMC is still being manufactured and sold at the time of writing this review. All devices are still used to this day and are still in circulation.

For more information regarding the hardware of both Kinects and the LMC, readers may consult Section 3.1 and Section 3.2. The information found in this whole section on the devices should be considered as an introduction or a summary. The information is detailed in later sections.

### 3.1. Kinect Devices

**Figure 4:**
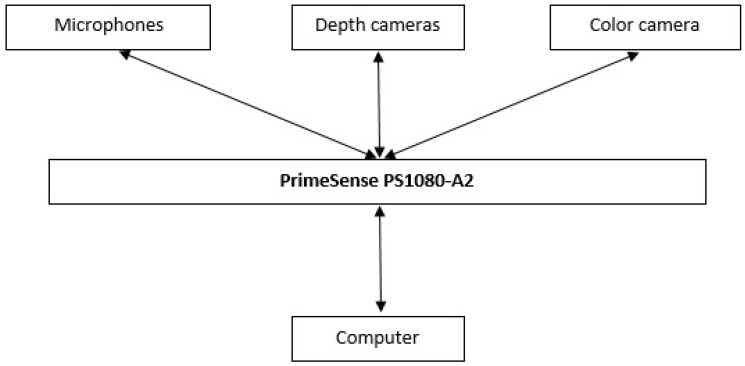
Flow of data between parts of the Kinect v1.

Both Kinect devices can track the whole human body. Both feature two depth cameras which means that they combine two types of techniques for depth mapping: focused and stereo. Focus means that when objects are further from the device, they become blurrier. However, the Kinects use astigmatic lenses with different focal lengths on the x and y axes to improve the accuracy [18]. With stereo, it calculates depth from the disparity [19]. In addition to the depth cameras, the Kinect devices both have a microphone array which consists of four microphones. The microphones are equipped with a multichannel echo cancellation feature and can also track sound position. The microphones can suppress and reduce noise. Aside from these, the hardware of both Kinects are different from each other in other aspects. The Kinect v1 has a 64 MB DDR2 SDRAM inside and uses a PrimeSense PS1080-A2 chip which processes data before transmitting [20]. This can be seen in Figure 4. Its infrared emitter has a 60 mW laser diode and functions on a wavelength of 830 nm. The Kinect v2 has a Samsung K4B1G1646G 128 MB DDR3 SDRAM and features a Microsoft X871141-001 chip instead of the PrimeSense one [21].

**Figure 5:**
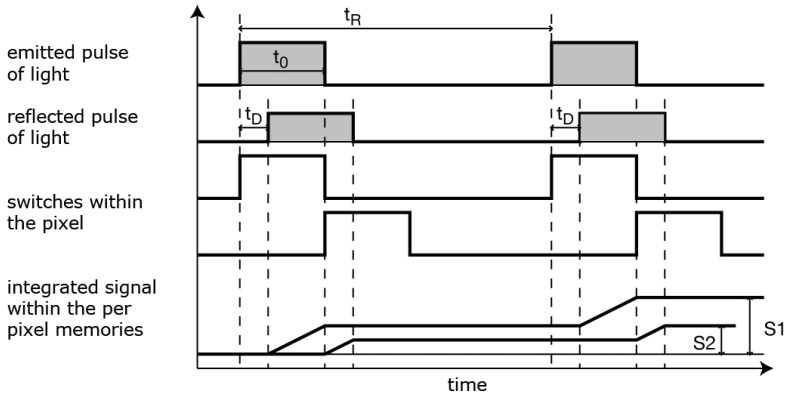
Illustration of the Time-of-Flight method.

The Kinects mainly function as depth sensors. In contrast to their hardware, both Kinects are similar in the way that they use depth mapping. Their depth mapping techniques, however, are different. The Kinect v1 uses its infrared emitter to emit infrared dots and can calculate distances based on their distortions [22]. The Kinect v2 uses a Time-of-Flight (ToF) method which measures the speed of light to calculate the distance [23]. The latter can be seen in Figure 5. More information on both depth mapping methods can be found in Section 6.1.1, where their positive and negative attributes are presented.

There is another way for motion tracking: Microsoft released Software Development Kits (SDKs) for both Kinects which feature a so-called “skeleton stream”. This skeleton stream allows the developers to track the joints of the user in real-time. More information can be found in Section 6.1.2. 

### 3.2. The LMC

The integrated circuit (IC) of the LMC is a Macronix 25L320E which stores the USB controller’s firmware in 32 Mbits. It allows for both USB 2.0 and USB 3.0 connections routed into different parts of the IC. The IC of the USB controller is a CYUSB3014-BZX by Cypress Semiconductor (San Jose, CA, USA) and the device also features a P-Channel MOSFET with an ID of FDD6685 made by Fairchild (South Portland, ME, USA). The LMC itself is manufactured by Sunny Optical [24].

**Figure 6:**
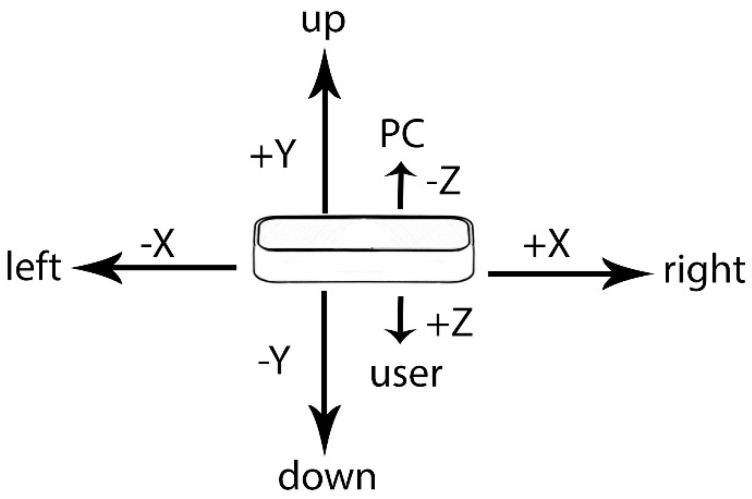
The different axes of the LMC.

The device has two cameras and three infrared LEDs inside it which can detect infrared light. Due to its two cameras it creates a grayscale stereo image from the infrared light data, but it does not map using its depth cameras like the Kinect. Instead, the device uses algorithms to calculate the data of the hand from the raw sensor data. This raw data is made up from infrared brightness values and calibration data to fix lens distortions. Its motion tracking range is between 2.5–60 cm according to the official blog of the manufacturer and Wright et al. [25], but since the Orion beta version came out in 2016 it has been expanded to 80 cm [26]. An illustration of the LMC’s hand tracking and Field of View (FoV) can be seen in Figure 6. The LMC can capture skeletal data of the hand as well. It is a software method made possible by its official application programming interface (API) as of version 2 [27]. The skeletal data can be acquired in a 3D space [28] which is—in a way—similar to the Kinect devices as their data can also be acquired in a 3D space. This means that the Kinects and the LMC can see the depth of the image, but with different methods and all have a skeletal motion tracking feature, where the frames of the joints can be accessed real-time.

However, the LMC suffers from latency issues. This has been evidenced by Silva et al. in 2013 [29]. The test used Digital Musical Instruments (DMIs)—pianos to be exact—written in Java language to measure the latency of the LMC. In their study, all three performance sets of the LMC has been tried – these are available in the LMC SDK. The approximate frame rate per second and the delay of these performance sets can be seen in Table 2. However, in the study the delay was 71 ms instead of 5 ms in High Speed mode which raises questions: are there other factors which increase latency? In the blog hosted by the manufacturers of the LMC, it is mentioned that there are hardware and software factors regarding the latency [30,31]. This can be seen, for example, in the fact that by switching from the USB 2.0 port to the USB 3.0 port the frame rate per second can by multiplied by one and a half. Even the display monitor could be at fault when talking about latency. In that study, they also mention that the acceptable latency should be less than 20 ms, so using DMIs should only be advisable with a slow rhythm. Also, a tracking problem surfaced when conducting the latency tests: when the fingers are too close to each other while playing the piano, the LMC could not differentiate between the fingers. In that case, not all or false musical notes were played depending on the position of the fingers. 

## 4. Motion Tracking State of the Art

Studying of the literature of motion tracking is needed to give context and to answer our RQ. Motion tracking as a whole is however a vast field of research. The sensors used in motion tracking can be classified into multiple categories according to Zhou and Hu [32]. According to them, the three main tracking categories are “Non-visual tracking”, “Visual-tracking” and “Robot-aided tracking”. All three of these main categories have multiple subclasses. Also, in their classification study, they did a survey on the use of sensors in the field of rehabilitation. They surveyed multiple sensors from all three main classes, but came to the conclusion that these sensors are not patient-oriented, they do not allow home use and are expensive. In contrast, both the Kinect and LMC are low-cost sensors and allow home use if the therapist allows it. However, since the survey was made in 2008, it has to be noted that the Kinect sensor and the LMC did not even exist at that time. 

Based on the mentioned survey of Zhou and Hu [32], both the Kinect sensor and the LMC can be classified into the marker-free visual-based category: According to the authors the sensors that are classified in this category have high accuracy, high compactness, inefficient computation, low-cost and their only drawback is occlusion. 

As both are marker-free visual-based sensors, the Kinects and the LMC can only track motions which happen in front of them (meaning that the movements happen in their FoV). To increase this FoV, an indoor localization study was done with Kinects [33]. In that study, the authors connected three Kinect devices at different angles. Depending on the angle of the user, one of the three Kinect devices started to track the user. The Kinects were selected by the Bivariate Gaussian Probability Density Function and the Maximum Likelihood Estimation methods. They concluded that this is not only precise, but a low-cost substitute for more expensive sensors.

Also, because of the marker-free visual-based classification and—possibly—their low prices, both sensors are used in multiple fields and were the targets of multiple literature reviews and other assessments in the past. In 2011, while the Kinect was still young, its educational usefulness was assessed [34], which yielded skeptical, but positive results. In 2014, Bacca et al. [35] reviewed the trends in AR and found that the demand for educational AR games for the Kinect was increasing while mentioning that its object tracking should be improved algorithmically.

In 2014, Hondori and Khademi [36] studied the clinical and technical impact of the Kinect sensor while comparing it to the LMC, Asus Xtion Pro Live and Intel Creative. Also, according to them, the number of papers indexed by PubMed assessing the Kinect increased drastically from 2011. They concluded that the Kinect is useful in medical applications. Reis et al. [37] concluded that most studies only include upper limb rehabilitation and most studies focus on serious games with the Kinect to make rehabilitation and education fun and motivating. In 2015 Da Gama et al. [38] also concluded that motor rehabilitation is possible, but the skeleton tracking of the Kinect should be improved.

There are other studies regarding the Kinects which do not cover precision and accuracy, but rather focus on their use and suitability in multiple fields: virtual laboratories for education [39], helping children with special educational needs [40], measuring and improving mild cognitive impairment [41], improving motivation [42], exercise gaming [43], establishing a gesture controlled interface for people with disabilities [44], assessing game performance of people with physical disabilities [45], studying navigational issues of people with movement disorders in a virtual environment [46] and other, virtual reality therapies [47,48]. 

Multiple reviews exist for the LMC as well. The most recent was in 2018, where Bachmann et al. reviewed [49] 3D HCI with a focus on the LMC. Though the study mostly contain information about 3D HCI, information about the hardware side of the LMC is also contained therein. Similarly to the Kinects, the LMC can be found in other studies regarding its use and suitability. Since it tracks hands, multiple studies have been conducted for sign language recognition: American [50,51], Arabic [52,53,54], Australian [55], Greek [56], Indian [57], Israeli [58] and Mexican [59]. It can also be found in education [60], in studies regarding upper limb rehabilitation [61,62,63], wheelchair maneuvering [64], robotic arm navigation [65,66]. Also, in [67] a phantom tumor was removed. Its conclusion is that in some cases, surgery with the LMC could be possible and suitable. However, haptic feedback should be included and more research may be necessary in this field.

A research on the trends of HCI was done in 2018 [68], concluding that these devices will make the standard keyboard, mouse and other traditional input devices obsolete. When dealing with simple gestures, mainly in the field of physical rehabilitation for people with disabilities, the Kinects and the LMC already replaced the mouse. This can be seen in the aforementioned studies. However, when doing more complicated gestures, the mouse is still superior. In contrast, the manufacturers of the LMC have the aim to replace the mouse in all hand movements. At the moment, they are working on a device called North Star [69] which is an augmented reality headset combined with the LMC. It is possible that after release, it will make the mouse and keyboard obsolete.

It should be noted that the studies involving these devices work with their own, private datasets. There is, however, a public example dataset for the Kinect v1 provided by Microsoft [70] which can be used for testing purposes. For the LMC, no public example datasets exist, but example applications are available [71].

To summarize, with the studies reviewed in this section, the authors presented different uses of the devices. The authors believe that these studies are socially important as well, and could raise the awareness of the readers. Also, the studies provided positive results or conclusions, making the devices suitable in these fields of research. This gave context to our RQ.

## 5. Discussion: Comparisons to Other Sensors

From this section onwards, the technical side of the devices is reviewed, starting with device comparisons found in the literature. In Section 5.1, the Kinects are compared to several other devices which use markers or are marker-free, respectively. In Section 5.2, the LMC is compared to other, wearable devices. In the following subsections, devices in the same price range and more expensive ones are compared with the Kinects and the LMC. All devices function well in their respective fields of research, whether they are more expensive or in the same price range. However, it is important to determine whether the cheaper sensors can achieve the same or similar results for less price. 

### 5.1. Kinect Sensor

Gonzalez-Jorge et al. [72] compared the Kinect v1 to the Asus Xtion. Similarly to the Kinect v1, the Xtion is also equipped with a depth camera [73] and works in a similar way. They project infrared dots and use their depth camera to calculate depth based on the mentioned infrared dots. Both sensors use the PrimeSense infrared measuring unit. In contrast to the Kinect v1, the Xtion does not require an external power supply: It connects to the computer through the USB port and this supplies it with power. In the study artefacts were measured at the angles of 45°, 90° and 135° but the angles did not affect accuracy and precision in the case of both sensors. The sensors did not produce an image at the range of 7 m. According to the study of Gonzalez-Jorge et al., both sensors behave in a similar way (due to their use of the same PrimeSense infrared measuring unit). The sensors could be used for multiple applications if the required tolerance is not strict. 

Breedon et al. [74] did a comparison between the two Kinect versions, the Intel RealSense SR300, Xtion Pro Live. The latter did not offer many improvements over the Kinects, but it has a better depth camera. Its depth camera resolution is 640 × 480. This is an improvement on the depth camera of the Kinect v2 with its resolution of 512 × 424. The Xtion Pro Live sensor was also discontinued like the Kinects. In 2018, a new version of the Intel RealSense, called the D415 has been released. Carfagni et al. [75] compared this device to its predecessor and to the Kinect v2. The raw data of the D415 provides less probing form errors, less probing size errors, less sphere spacing errors and less flatness errors than the raw data of the SR300 and the Kinect v2. In the study, it was concluded that the Intel RealSense D415 can be used as a low-cost device. It can be used in motion tracking, in gesture recognition and in other, 3D-scanning applications as well. Since all mentioned devices use depth mapping, a brief comparison is provided in Table 3.

Romero et al. studied [76] if the Kinect v1 sensor could replace the Polhemus Liberty Latus wireless system [77] while investigating the motor skills of children with Autism Spectrum Disorder (ASD). They came to the conclusion that in some ways it can substitute the Liberty Latus and in some ways it cannot: the Polhemus Liberty Latus gives a more accurate measurement with its electromagnetic (EM) field position and rotation mapping and in this regard is superior to the Kinect sensor. The Polhemus Liberty Latus is more suitable for measuring small scale, high precision tasks than the Kinect. On a large scale, however, if multiple limbs or whole-body tracking is used, the Kinect gives better results and its data is easier to use.

Sun et al. [78] found out that gesture recognition with the Kinect is possible by using its color camera along with surface electromyography (sEMG). Both sensors were used at the same time, fusing the acquired data. On the Kinect side, they used Fourier transformation and a characteristic line method and modeled the data on histograms. Also, because the color camera is used, the noise had to be filtered from the video. To achieve this, polygonal approximation, then a Douglas-Peucker (D-P) algorithm were used. Before the test, fifty training samples were collected and another fifty were collected as silhouette samples. Two hand gestures, four wrist gestures, and four finger gestures are tested, twenty of each gesture in five different groups (which means the total of 100 for each gesture). With the sEMG averaging in 60-65 gestures and the Kinect averaging in 80-90 gestures, they concluded that the Kinect is superior to sEMG.

A firearms training simulator has been developed by Bogatinov et al. [79] to replace more expensive existing simulators on the market. The gestures inside the application has been created with the Flexible Action and Articulated Skeleton (FAAST) toolkit [80]. They propose that their simulator is better and cheaper than other military simulators such as MINT-PD [81] when used with nine calibration points and when the player is 2.5 m away from the Kinect sensor. Using MINT-PD is more expensive than using the Kinect. That is because it requires the set-up of a controlled environment with a laser and a laser-tracker, a microphone for speech recognition and a tablet for special input.

As a side note, not just human motion tracking is available with the Kinect sensor, but environmental tracking as well: Rosell-Polo et al. used the Kinect v2 for agricultural outdoor applications [82]. Normally, in this field people use Light Detection and Ranging (LiDAR) sensors which consist of Terrestrial Laser Scanners (TLS) or Mobile Terrestrial Laser Scanners (MTLS). They found that the Kinect v2 sensor is similar to both TLS and MTLS due to its color and depth cameras. However, the Kinect v2 comes with a shorter range and a narrower FoV than LiDARs. In the study, the authors combined the Kinect v2 sensor with a real time kinematic Global Navigation Satellite System (GNSS). The authors of the mentioned study used different FoVs with different sampling rates: 5.15 Hz with a single-column FoV, 0.75 Hz with partial FoV, and 0.15 Hz with full FoV. Naturally, 5.15 Hz results in the best output, however there could be up to 1.5% of errors. In short, they achieved a low-cost and effective substitute for LiDAR sensors. In another study by Keightley and Bawden [83] it can be seen that the ILRIS 3D LiDAR sensor can be used for environment tracking, thus giving this paper a LiDAR sensor to compare the Kinect v2 with.

Table 4 presents a summary of the comparisons between the devices. In the table four columns can be found: the name of the device, its mapping type, its sampling rate and its market or used price at the writing of this review. As suspected, the Polhemus Liberty Latus, the sEMG and the ILRIS 3D are extremely expensive. Regarding cost, there is no information available on the MINT-PD as it has been developed for the military. Also, it has to be noted that the first four devices in Table 4 were discontinued at the time of writing this review. This means that only used ones are available on the market, thus its price might vary from seller to seller.

### 5.2. Leap Motion Controller

Naturally, the LMC has only been compared to sensors which can track hands. The first comparison was done with the Optotrak marker which is often used as the golden standard as it has errors around 1 mm. The Optotrak marker strobes the user and can be used wired and wirelessly [93]. This comparison was done in the study of Tung et al. [94]. The LMC had high degree of correlation with the Optotrak marker, specifically 0.995 on the horizontal axis and 0.945 on the vertical axis. Also, the finger accuracy of the LMC was 17.3 mm with a standard deviation of 9.56 to the Optotrak. In this study, the accuracy of the LMC was also assessed with the error of 17.30 mm. See Section 6.2 for further information on accuracy and precision.

Another comparison was done in another study to the Myo Armband which uses Bluetooth Low Energy frequency (2.402–2.480 GHz) connectivity with a sampling rate of 200Hz. Chen et al. compared the LMC to the Myo Armband through the use a virtual reality application made inside the Unity game engine [95]. Though there are no precision and accuracy data was available in that study, a comparison and analysis were done through controlling the game. They measured the success rate of the users during playing the game with the following method: if the user falls from the level, it is considered as a fail. With the Myo Armband, the total number of falls was 84 and with the LMC the number during testing was 74. This leads to the conclusion that either the LMC is more accurate and precise than the Myo Armband or it is easier to use. The latter is also concluded by that study where 17 participants stated that the Myo Armband was the hardest “game controller” to use, and 10 participants stated that the LMC was the hardest to use. However, they also concluded that there is little research available on continuous movements that require high precision.

Breedon et al. [74] compared the LMC and the Creative SENZ3D. The SENZ3D has multiple advantages over the LMC: it can be controlled by voice as it has a dual array microphone. It can also detect head movements and features facial recognition additionally to hand tracking. This allows it to capture images of head contours. While it has some improvements over the LMC, it has some new limitations as well: Only one of its cameras can be used or only the voice capture function can be active at a time. It cannot use both of these features at the same time. At the time of writing this review, it is discontinued. Akin to the previous subsection, Table 5 presents a summary of the compared devices. The columns are extended by one, named Connectivity, which details how the device is connected to the computer. 

## 6. Discussion: Accuracy and Precision

After the device comparisons in the previous section, this section deals with their accuracy and precision when sensing depth. Also, since the authors believe that gesture recognition is one of the most important uses of these devices, existing algorithms are presented as well. The accuracy of these algorithms are also assessed. When choosing a sensor for a task, the authors believe these are the most defining factors. 

This section is made up from three subsections: studies about the Kinects, about the LMC and about using them together. The Kinects subsection is broken into two subsubsections, containing studies about their depth cameras and skeleton streams, respectively. The LMC subsection is not broken into two subsubsections like the Kinect. This is due to most research only deal with its skeletal data as the raw data is strongly distorted due to the wide field of view. The last subsection deals with researches which use all devices.

### 6.1. Kinect Sensor

When talking about the accuracy and precision of the Kinects, it has to be mentioned that two types of data can be extracted from the Kinects. The first is simply the raw data returned by the depth camera. Several studies have been done with raw data and multiple applications have been developed to extract and analyze the data. These are mentioned in Section 6.1.1.

The other type is called the skeleton stream, but it also uses the depth camera of the Kinect. It is basically a “software method” of returning the depth data as it is part of the SDK made by Microsoft. With the help of the skeleton stream, the Kinect can recognize joints in the human body and return real-time motion tracking data. It uses the mean-shift clustering algorithm to calculate modes of probability distributions to classify body parts into a virtual skeleton [18]. When using the skeleton stream, Microsoft recommends a range of 1.2–3.5 m between the Kinect and the user. It is mainly used for gesture recognition in medical applications, a few of them were mentioned when dealing with the state of art. In Section 6.1.2, the skeleton stream is reviewed.

#### 6.1.1. Depth Sensor

Wassenmüller and Stricker [99] compared the depth camera of the Kinect v1 to the Kinect v2. The Kinect v1 contains an infrared emitter which projects infrared dots into the environment and calculates the depth based on the distortion of the infrared dots. The Kinect v2 has a ToF camera which projects infrared light into the environment and calculates the depth of the scene by measuring the speed of the infrared light back and forth. In the study, a set of 300 depth images of the same environment was captured with a camera. Both Kinects change their depths regarding the sensor temperature. The mean depth of Kinect v1 decreases to less than 2 mm. The seen distance of Kinect v2 increases to 20 millimeters after using it for 16 minutes. However, when its 5 V- DC fan (labeled U40R05MS1A7-57A07A [21]) turns on, it decreases to 3 mm and then increases slightly when the fan starts to rotate with the same speed, so it is advisable to turn on the Kinect v2 16–20 minutes before using it. When the distance increases, the Kinect v1 has less accuracy and precision when detecting depth, with the offset increasing exponentially: at 0.5 m away from the sensor the offset is below 10 mm, but at 1.8 m away the offset can be more than 40 mm. With the increasing distance, a stripe pattern appears on the depth image of the Kinect v1 sensor. The number of stripes also increases with the distance. At different distances, the precision of the Kinect v2 decreases, but the accuracy stays the same with a –18 mm offset where the central pixels are all the same, only the corner pixels could be incorrect. If the plane is flat, the precision is higher for the Kinect v1. However, if the plane is not flat or if there are discontinuities then the precision is less with the Kinect v2 and flying pixels could appear. Flying pixels are not present with the Kinect v1 as it is not a ToF camera. Environment color also affects depth estimation with the Kinect v2: black colors have 10 mm more depth value. Also, multipath interference is present with the Kinect v2 which means that concave geometry is represented with bulges. 

A study of accuracy and precision was made by Gonzales-Jorge et al. [100]. They measured an artefact at multiple angles with both Kinect sensors. The angles were 45°, 90°, 135° but the angles did not affect accuracy and precision as previously mentioned in a study when comparing the Kinect sensor to the Xtion sensor. These angles were measured at multiple distances where 1 m was the closest distance and 6 m was the largest. The Kinect v1 sensor can sense up to the range of 6 m while the Kinect v2 sensor was only capable up to 4 m. Even though the range of Kinect v1 sensor is larger than of the Kinect v2 sensor, it is less accurate. Aside from accuracy, the precision worsens with both sensors as the range increases, but the precision differences are always less for the v2 sensor than for the v1 sensor. Even at the range of 1m the precision of the Kinect v2 sensor is better compared to the Kinect v1 sensor: the precision of the Kinect v1 decreases with the second order polynomial when increasing the range. While no mathematical behavior was found for precision values for the Kinect v2, it is possible to give similar results with an equation. The precision values are defined in Equation (1) for the Kinect v1 and in Equation (2) for the Kinect v2:

y_Kinect1_ = 2.0399Z^2^ − 2.016Z + 2.0957 (1)

y_Kinect2_ = 0.5417Z^2^ − 0.885Z + 2.7083 (2)

According to Khoshelham et al. [101], the error of depth measurements with the Kinect v1 can increase quadratically to 4 cm at the range of 5 m while the depth resolution decreases quadratically. The Kinect v1 sensor also has a standard deviation of approximately 15 mm in its depth accuracy. The conclusion is that depth measurements should be done between 1–3 m. 

Similarly, the Kinect v2 was studied by Yang et al. [102] with the aim to improve its depth accuracy. According to them, the average depth accuracy error is less than 2 mm until the user is 3 m away from the device. Between 3 m and 4 m the average depth accuracy error is between 2 mm and 4 mm. If the user stands farther away from the sensor, the average depth accuracy error is more than 4 mm. This is only true of the user stands directly in front of the Kinect 2. If the user steps sideways, then the average depth accuracy error increases.

Interestingly, despite the “actual minimum measuring distance”, Chan et al. [103] managed to calculate the volumes of eggs at least 70 cm away from the Kinect v2 sensor, possibly due to their size. They placed the Kinect v2 sensor in four different positions in multiple 45° angles facing the eggs. Regarding the distance to the eggs, they found out that the best distance from the Kinect v2 is between approximately 70–78 cm and 74 cm gives the best results. Regarding the positions, they concluded that without shear parameters the deviation of volume estimation is between ±1.74 mL and ±3.62 mL; and with shear parameters it is between ±0.05 mL and ±9.54 mL. Naturally, the differences also depend on the size of the egg. In terms of accuracy, 84.75% was the worst they could find and 97.86% was the best. The mean accuracy is 93.3% which can be considered good.

There is more conflicting information about the range of the Kinect v1 and v2. Kadambi et al. [104] did a study on the specifications of the Kinects. However, they concluded that the depth of the Kinect v1 can be 0.4–3.0 m or 0.8–4.0 m and the depth of the Kinect v2 can be 0.5–4.5 m depending on environmental conditions, though these depth ranges have not been tested in the study. The data collected from this and the previous studies can be seen in Table 6. There are three types of distances in the table: the specified distances which are the ones mentioned in the previous study, the recommended distances by the manufacturer and the tested distances which have been proved and tested in previously mentioned studies.

Bragança et al. performed a study [108] to see how precise the Kinect sensor is. For that purpose, they developed a 3D scanner system which was arranged with four Kinect devices. They did manual anthropometric measurements with a simple measuring tape and compared the results to their 3D scanner system. Out of the four Kinects, two were placed in front of the volunteers and two were placed behind them at the height of 50 cm and 140 cm, respectively. The Kinect sensors were 125 cm away from the participants in each direction. The study had 37 participants of different age, height and weight. When measuring the ten body parts, they used six different evaluation parameters. The parameters were:

- Technical Error of Measurement (TEM)
- Relative Technical Error of Measurement (%TEM)
- Intraclass Correlation Coefficient (ICC)
- Reliability Coefficient (R)
- Standard Error of Measurement (SEM)
- Coefficient of Variation (CV)

While there were some small deviations in centimeters when comparing both methods, they found out that the Kinect sensors is a viable solution for lower levels of precision. It should be noted, that not only the hardware, but the software is equally important for precision. Their results are summarized in Table 7 alongside the results of Mankoff and Russo [109] who concluded that the actual distance to an object is less than the distance that the Kinect sees. 

Also, gesture recognition is possible with the depth sensor. Chikkanna and Guddetti [110] used Hidden Conditional Random Field (HCRF) which learns a set of latent local variables. The variables are conditioned on local features. In the study they developed an algorithm for Indian sign language recognition with the Kinect v1 where they recorded 650 gestures beforehand. With HCRF 93.2% and 95.2% of the gestures were recognized in real-time and not real-time, respectively.

#### 6.1.2. Skeleton Stream 

The skeleton stream is available via the SDK made by Microsoft and it is a “software method” to access depth data in real-time. Both Kinects feature the skeleton stream, though there are small differences. In Table 8 a brief comparison of the two skeleton streams can be seen.

According to Livington et al. [111], the skeleton stream can be acquired with the Kinect v1’s SDK between 0.85–4 m in contrast with the range recommended by Microsoft. Outside of these bounds the sensor won’t return data. They also found out that when using the Kinect v1’s SDK, this also applies to the depth data. This contrasts with the 6 m mentioned earlier in this review where the researchers did not use the SDK. In their study, they measured the noise of the skeleton stream. At 1.2 m away from the sensor, the noise was 1.3 mm with a standard deviation of 0.75 mm and at 3.5 m away, the noise was 6.9 mm with a standard deviation of 5.6 mm. In the Kinect v1, the average noise also changes from dimension to dimension: x = 4.11 mm, y = 6.2 mm and z = 8.1 mm. They also found out that the right wrist and hand gave the most noise. Accuracy of the skeleton stream has been tested as well, averaging at 5.6 mm with a standard deviation of 8.1 mm and no difference was found when taking the dimensions into account. Another interesting fact is that when one person uses the Kinect v1, the error in accuracy is 1.4 mm. In contrast, when two people uses the device, the error increases to 1.8 mm. With three people, the error becomes 2.4 mm—even though the Kinect v1 is only able to track two people. The mean latency of with one skeleton is 146 ms and with two skeletons is 234 ms but these data largely depend on the computer’s hardware configuration, and other, simultaneously running applications.

Otte et al. [112] discovered that the range of skeleton steam via the Kinect v2’s SDK is between 0.5–4.5 m when researching its accuracy with young healthy adults. This range slightly larger than the range with the Kinect v1’s SDK. In the study, the data from the Kinect v2 was not smoothed. They concluded that the Kinect v2’s skeleton stream yields adequate results, however the sensor has a harder time differentiating between the ground and the feet of the user.

In the study of Reither et al. [113] upper extremity movements, mainly of the shoulder joint were measured using the skeleton streams of both versions of the Kinect and compared to a Video Motion Capture (VMC) system. The data was filtered with a fourth order Butterworth filter at 6 Hz. Both Kinects had good reliability, though the Range of Motion (ROM) was underestimated by the Kinect v1 for reaching type of movements and overestimated for angular movements. The Kinect v2 performed well for forward reaching type of movements, however its performance for the side movements was not as good. Angular movements were overestimated by the Kinect v2. Even though the ROMs measured by the Kinects were different from the VMC, both Kinects measured movement patterns very well. Transformation of the Kinect’s skeleton data could make the data similar to VMC’s data, allowing it to be used in medical applications.

Similarly to manual measurements in the previous subsection, the Kinect v1 was compared to a magnetic tracking system and a goniometer by Huber et al. [114] where joint angles were tested. The study included frontal and side views. The mean difference from the goniometer was between −4.1° and 17.8° during the gestures, and the mean difference from the 3D magnetic tracker was between −24.2° and 20.6°. They concluded that the Kinect is reliable when the shoulder joints are not occluded.

Elgendi et al. [115] made a study with ten subjects who all did three types of gestures: slow, medium and fast. The gestures were done with a shift of 45° to the right make sure that the body does not interfere with the hand movements. They used a low-pass filter which is a first order Butterworth low-pass filter with a cutoff frequency of 2 Hz to reduce the environmental or bodily noise from the skeleton stream. However, even without the low-pass filter they concluded that the hand is the most reliable for detecting speed with the lowest error rate of 9.33%. With the low-pass filter, the lowest error rate is 8%.

To increase the recognition with skeletal tracking the Extended Body-Angles Algorithm (E-BA-A) [116] were used in the study of Gutiérrez-López-Franca et al. in 2018 [117]. During the study they found out that the number of the used joints in the body during measurements affects the number of errors. Their measurements included:

- Global movements—where the whole body is used
- Bounded movements—where the movement only use a subset of the whole body
- Symmetric movements—where it is enough to measure “one half” of the body

According to them, global movements produce less error, however requires more computational power. Bounded movements have more errors, since the position of neighboring joints can affect the joints next to them. When using this method, it required less computational power than global movements. The results of the study were:

- With the “Specialized Body Parts Analysis” method: They used three different strategies with tree different bounds to calculate the exact rate of correct movement prediction with four different movements, two of which can be done with each half of the body. Using only the arms or legs gave the worst results with a 52.45% as it could not track the legs almost every instance, using the whole body gave better results with a 92.42% and interestingly when using only the arms or legs with the trunk gave the best results with 97.37%.
- With the “Stricker Restrictions for the Most Easily Detectable Activities” method: This method tries to improve on the postural coincidences where movements are similar and sometimes inferred by other joints. To improve on this, some restrictions had to be applied by introducing a barrier value, a minimum limit of prediction which also had to be tested multiple times to see if they are too strict. After selecting the values which are sufficient for them, they concluded that the arm movements got good results with a 92% and 93.6% accuracy, and the leg movements got 22.4% and 24.8% respectively which are great improvements to the first method.
- With the “Combination of Body Parts and Stricter Limits” method: This method is the combination of the two. This method gives the best results, and they can be achieved by using only the arms or legs with the trunk. It has a 96%–100% accuracy rate for the arm movements and 92.8%–96% for the leg movements.

### 6.2. Leap Motion Controller

When the LMC was in its preliminary stage, in 2013 a study by Weichert et al. [118] was made to assess its accuracy with the aim to increase its effectiveness in HCI. To achieve the best result, the study was done with an industrial robot with a position error below 0.2 mm. They concluded that an accuracy of less than 2.5 mm is possible, averaging around 1.2 mm. The standard deviation was below 0.7 mm per axis when moving. For example, when drawing a sine wave, the standard deviation was below 0.7 mm on the x-axis, below 0.5 mm on the y-axis and below 0.3 mm on the z-axis. Therefore, in cases when the motion path is important, the LMC should be used as according to them, this could not be achieved with the Kinect sensor as it is not as accurate. In another study, when comparing the LMC to the Optotrak marker, they concluded that the position error of the accuracy was below 17.30mm which is much worse [94]. It is possible that this error is due to it being from human data in contrast to the data from the industrial robot. However, the official specification of the LMC states that its accuracy is ± 0.009906 mm [119].

In 2014, another study [120] was made to assess the LMC’s accuracy for static and dynamic tracking. A plastic arm was used in the study. With static tracking, the standard deviation was always below 0.5 mm. In some cases it even reached less than 0.01 mm. With dynamic tracking however, the LMC has an inconsistent performance: The accuracy significantly drops by −5 mm when the user is more than 250 mm away from the sensor on the x-z plane. In the study they also concluded that the inconsistent sampling frequency makes it difficult to synchronize with other devices.

The speed of the Dynamic Time Warping method was tested. Vikram et al. [121] proposed a new method of handwriting: With the LMC, they tracked the fingers of the user to simulate writing of text. To look for similarities, Dynamic Time Warping (DTW) method was used, after optimizing it for real-time and after building a dataset of letters with 26,000 recordings and words with 3000 recordings. In the study, they found a handwritten word “new” with DTW in 1.02 seconds.

In 2015, Sharma et al. [122] proposed a method for number recognition: The user moves their hand in front of the LMC and based on the form of the gesture, a number is created by the application. Before testing, they taught five sample gestures per number. They achieved an average classification rate of 70.2% with the Geometric Template Matching Algorithm. Two years later, another method was proposed by Zeng et al. [123] which uses deterministic learning with twofold and tenfold validation cycles. Their number recognition rates were 94.2% and 95.1%, respectively, with the two cycles. 

In 2016, Jin et al. proposed a method [124] for two LMC devices with the aim manipulate objects on a table with a robotic hand. They use two LMC devices because according to them, the LMC is best in high precision mode when the palm of the hand rotates less than 60°. Over the said number, the gesture is susceptible to occlusion. During the study they used three different setups for the two LMC devices. A face-to-face setup, an orthogonal setup and a 120° angle setup:

- Face-to-face: It helps when the palm does a 180° rotation. 90° is still susceptible to occlusion.
- Orthogonal: It helps with a 90° rotation. In this setup, the 180° rotation is susceptible to occlusion.
- 120° angle: This setup was considered the best—and mainly used—in the study. If the palm is under 180° it provides the best results, otherwise both sensors have a worse recognition performance.

According to the study, the bottom sensors gave the worst results with every tested gesture. When the palm faced upwards the results could be as low as 6.7%. In contrast, with the side sensor the lowest result was 63.3% for the same gesture. When using both sensors the lowest result was 73.3%. Side movements were considered the best, 63.3%, 86.7%, 90% using the bottom, side and both sensors respectively.

Also, in 2016, a dynamic hand gesture recognition method was developed [125]. In the study they used two different datasets with the Hidden Conditional Neural Field (HCNF) classifier to recognize gestures. The HCNF is the extension of the HCRF classifier with gate functions of neural networks. During the study, one dataset produced an accuracy of 89.5% and the other 95%.

In 2017, Li et al. [126] reported a hand gesture recognition algorithm for post-stroke rehabilitation. This algorithm has a training phase and a test phase. It uses the Support Vector Machine mathematical method and the K-Nearest Neighbors classifier. With the former the algorithm achieved an average accuracy of 97.29% and with the latter it achieved 97.71%.

Mantecón et al. studied the precision of the LMC using only its infrared imagery [127]. They used the Depth Spatiograms of Quantized Patterns (DSQP) feature descriptor to achieve characterization of hand gesture information. After that, the Compressive Sensing-based dimensionality reduction algorithm was used as the data vector was too large. Lastly, SVM solution was used for classification. With this software method and using the raw data of the device, they achieved a gesture recognition rate of 99%–100%.

### 6.3. Using the Two Devices Together

In Section 4, the authors determined that the LMC is popular for sign language recognition. However, in the study of Marin et al. [128], not only the LMC, but the Kinect is also applied and compared. According to them, the LMC has an accuracy of 80.86% of detecting the gestures, the Kinect has an 89.71% accuracy and when the two sensors are combined, the accuracy increases to 91.28%.

Penelle and Debeir proposed a system in their study with the fusion of the LMC and Kinect v1 [129]. The setup consists of the LMC placed on top of a desk in front of the user and the Kinect v1, also on top the desk but facing the user about 1m away from them. Since the sampling rate of the Kinect is 30 Hz, and the LMC was around 115 Hz in the study, both had to be calibrated and synchronized. For calibration they used the Corresponding Point Set Registration (CPSR) algorithm while working with the fingertips of the user. After calibrating and synchronizing, they compared data from both devices with the conclusion that the average for maximum error is always below 20 mm and the median error is between 5–10 mm in all cases. 

Lastly, a study was conducted at Stanford University [130] where a VR Angry Birds game was created with the fusion of the LMC and the Kinect v2. It should also be noted that there is some erroneous information regarding the Kinect v2 in that study. When talking about its range and FoV, the specifications given are those are of a Kinect v1. Since the study only talks about the user experience side of HCI, there are no new concrete measured data regarding both sensors. On the user experience side, however the users felt that they have worse controls when using only the Kinect as it is noisier than the LMC. Also, when executing slow throws in the game, the users felt that the LMC is more useful for slow, precise throwing calculations. In the study, the Kinect v2 was placed in front of the user and the LMC was placed on an HMD which was on the head of the user. This means that the sensors face each other. The authors of this literature review developed an application with the Kinect v2 and the LMC [131] and since the study at Stanford did not talk about the interference between the two sensors, it should be mentioned that when testing the application of the authors, it was found out that when the Kinect v2 and LMC is positioned towards each other (which is also the case with the study at Stanford), there is a possibility that the devices interfere with each other in a way that the tracking of the LMC becomes less accurate due to the IR signals from both devices.

Even though they are devices in different categories, since they have been used together, they should be compared on the hardware side as can be seen in Table 9. 

## 7. Conclusions

The human motion tracking state of the art is presented in this paper, establishing its use and classification methods while focusing on low-cost sensors, namely the two versions of the Kinect—for whole body tracking—and the LMC—for hand tracking. 

When analyzing the studies, it became apparent that all three devices are popular, however the authors concluded that the later Kinect v2 as not as widely used as the Kinect v1. Most studies—even in late-2018—use the Kinect v1. No study mentions why it is used more often. Cost issues are probably not the case as at the writing of this review the price of the two versions is the same. After the assessment of the two versions, the authors concluded that pros of the first version outweigh the pros of the second: the depth precision is higher, environment color does not affect depth estimation and it has a weak correlation to the temperature. When using the Kinect v2, more attention must be paid to the whole test environment than with the first version.

When comparing all three sensors, the authors came to the conclusion that the LMC is more accurate. While not reaching the exact accuracy specified in its official specifications, both its accuracy and precision are greater than those of both Kinects. Though the Kinects and the LMC are impossible to compare to each other as they have different functions, meaning that the two Kinects track the whole body, and the LMC only tracks the hand, however, for a method to actually compare the devices, the authors have a suggestion for future research: first, the size of the emitted infrared point-cloud of the LMC should be measured. Then, a similar measurement should be done for the emitted point-cloud of the Kinects. After that, a same-size surface of both point-clouds should be selected by researchers. Finally, the selected surfaces should be compared to each other, regarding their depth, accuracy, precision, etc. The authors believe that this is an interesting idea for future research regarding device comparison.

At the moment, however, it can be concluded that the Kinects are two of the most accurate low-cost whole human body motion tracking sensors available, while the LMC is one of the most accurate low-cost hand tracking sensors. This makes them suitable in multiple fields of research such as education, rehabilitation, gesture recognition, entertainment, etc. When doing tasks however, multiple researchers have expressed the desire for haptic feedback as they felt that using these devices would feel more natural if they had haptic feedback. Therefore, it may be an interesting and viable research area for future researchers.

The answer to our RQ is that these three sensors can replace more expensive sensors in the industry or on the market. While they are not inaccurate devices on their own, software methods exist to improve on them. For example, the Orion which was made by the same makers of the LMC had an increased measuring distance. Also, many algorithms exist made by researchers in the field to increase the gesture classification accuracy of these devices up to 99%–100%. Also, when using multiple sensors or when these devices are combined, their accuracy increases.

Sadly, little to no information on the required computational power was found in the researched articles. When assessing the accuracy of the mentioned algorithms, their required computational power is an important attribute. Especially, if a study is about using the sensors at home for motion tracking purposes.

While not focusing solely on the Intel RealSense sensors in the review, the authors believe that the Intel RealSense sensors have the possibility of becoming a new alternative when choosing low-cost whole-body tracking devices. At the moment, their price is about one and a half times more of the Kinects, but based on their hardware specifications they are worth the price. Due to the fact the Kinects are discontinued as of the beginning of 2018, it is possible that in a few years the Intel RealSense sensors will take over the mantle of the Kinects.

Still, the authors hope that this review helps the users choose from an “extended sensor pool” for their research works and projects. The authors hope that researchers will not only consider expensive sensors, but at the moment, they are increasing their decision-rate towards the Kinect devices and the LMC.

## Figures and Tables

**Table 1:** Search results for the literature review.

	Number of Results		
Keywords	Web of Science	PubMed	IEEE Xplore
Kinect review	74	36	48
Kinect accuracy	635	200	884
Kinect precision	105	4	128
Kinect skeleton	183	99	578
Kinect gesture recognition	240	30	727
Kinect medical applications	27	19	183
Kinect physical disability	22	22	24
Kinect education	67	77	183
Leap Motion review	12	8	6
Leap Motion accuracy	66	18	83
Leap Motion precision	19	7	20
Leap Motion gesture recognition	36	9	147
Leap Motion medical applications	6	7	34
Leap Motion physical disability	3	3	3
Leap Motion education	10	12	25

**Table 2:** Performance of the LMC with an USB 2.0 port.

Mode	Approximate Frame Rate/Second	Delay
High Precision mode	50 fps	20 ms
Balanced Tracking mode	100 fps	10 ms
High Speed mode	200 fps	5 ms

**Table 3:** Brief comparison of similar price range, whole body tracking devices.

	Kinect v1	Kinect v2	Xtion	Xtion Pro Live	Intel RealSense SR300	Intel RealSense D415
Color camera resolution	1280 × 720 at 12 fps, 640 × 480 at 30 fps	1920 × 1080 at 30 fps	640 × 480 at 30 fps	1280 × 1024 at 15 fps, 640 × 480 at 30 fps	1920 × 1080 at 30 fps, 1280 × 720 at 60 fps	1920 × 1080 at 60 fps
Depth camera resolution	320 × 240 at 30 fps	512 × 424 at 30 fps	320 × 240 at 30 fps	640 × 480 at 30 fps, 320 × 240 at 60 fps	640 × 480 at 30 fps	1280 × 720 at 90 fps
Depth technology	Infrared	ToF	Infrared	Infrared	Coded light	Stereoscopic active infrared
Field of view ^1^	57°H, 43°V	70°H, 60°V	58°H, 45°V	58°H, 45°V	73°H, 59°V	69.4°H, 42.5°V
Specified measuring distance	0.4 or 0.8 m–4 m	0.5–4.5 m	0.8–3.5 m	0.8–3.5 m	0.3–2 m	0.16–10 m
Connectivity	USB 2.0 or 3.0	USB 3.0	USB 2.0	USB 2.0	USB 3.0	USB 3.0 Type-C

^1^ H stands for horizontal, V stands for vertical FoV.

**Table 4:** Results of the reviewed articles.

Name	Mapping	Sampling Rate ^1^	Cost
Kinect v1	Depth (IR)	30 Hz	US$99.95 [84]
Kinect v2	Depth (ToF)	30 Hz	US$99.99 [85]
Xtion	Depth (IR)	30 Hz	€50 [86]
Xtion Pro Live	Depth (IR)	15 Hz	US$140 [87]
Intel RealSense SR300	Depth (Coded light)	30 Hz	€68.12 [88]
Intel RealSense D415	Depth (Stereo active IR)	90 Hz	US$149 [89]
Polhemus Liberty Latus	EM field	188 Hz or 94 Hz	US$12,500–US$60,000 ^2^
sEMG	Electrodes	800 Hz–1 kHz ^3^ [90]	US$25,000 ^4^ [91]
MINT-PD	Laser	No information.	Not available.
ILRIS 3D	Laser	2500 points/s	€16,000 [92]

^1^ The sampling rate is defined on the largest possible resolution of the device. ^2^ Cost depends on the number of sensors. ^3^ The most common use, up to 6 kHz is possible. ^4^ It is the cost of the BTS FreeEMG 1000. It is possible that there are less or more expensive devices on the market.

**Table 5:** Results of the reviewed articles.

Name	Mapping	Sampling Rate	Connectivity	Cost
LMC	Algorithmic	50–200 Hz ^1^	USB 2.0 or 3.0	US$80 [96]
Optotrak marker	Strobe	120 Hz	Wired/Wireless	Not available.
Myo Armband	Electrodes	200 Hz	Bluetooth	US$200 [97]
Creative SENZ3D	Depth	30 Hz	USB 2.0 or 3.0	US$79 [98]

^1^ The sampling rate of the LMC is 50–150 Hz according to [94], but in [29] it is 50–200 Hz.

**Table 6:** Kinect v1 compared to Kinect v2 with their technical specifications.

	Kinect v1	Kinect v2
Dimensions	27.94 cm × 6.35 cm × 3.81 cm [105]	24.9 cm × 6.6 cm × 6.7 cm [106]
Color resolution and fps	640 × 380 at 30 fps or 1280 × 720 at 12 fps	1920 × 1080 at 30 fps
IR resolution and fps	640 × 480 at 30 fps	512 × 424 at 30 fps
Depth resolution and fps	320 × 240 at 30 fps	512 × 424 at 30 fps
Field of view wide-angle lens	57° horizontal, 43° vertical	70° horizontal, 60° vertical
Specified min. distance	0.4 m or 0.8 m	0.5 m
Recommended min. distance	1.8 m	1.4 m
Tested min. distance	1 m	0.7 m
Specified max. distance	4 m	4.5 m
Tested max. distance	6 m	4 m
Active infrared	Not available	Available
Measurement method	Infrared structured light	Time of Flight
Minimum latency	102 ms	20 ms
Microphone array	4 microphones, 16 kHz	4 microphones, 48 kHz
Tilt-motor	Available, ±27° [107]	Not available
Temperature	Weak correlation	Strong correlation
More distance	Less accuracy	Same accuracy
Striped depth image	Increases with depth	No stripes on image
Depth precision	Higher	Less
Flying pixels	Not present	Present if surface is not flat
Environment color	Depth estimation unaffected	Affects depth estimation
Multipath interference	Not present	Present
Angles affect precision	No	No
Precision decreasing	Second order polynomial	No math. behavior

**Table 7:** Comparison of Kinect measurements and manual measurements.

	Kinect	Manual Measurement
Precision	Less precise	More precise
Measuring speed	Faster	Slower
No. of best measurements	Eight “best” results	12 “best” results
No. of worst measurements	Six “worst” results	Five “worst” results
Nearest measured distance	501 mm	500 mm
Farthest measured distance	5050 mm	5000 mm

**Table 8:** Skeleton stream comparison of both Kinect sensors.

	Kinect v1	Kinect v2
Max. number of tracked people	2	6
Available joints to track	20	25
Tested distance	0.85–4 m	0.5–4.5 m

**Table 9:** Comparing the Kinect v2 to the LMC.

	Kinect v2	LMC
Dimensions	24.9 cm × 6.6 cm × 6.7 cm	7.874 cm × 3.048 cm × 1.27 cm
Tracking hardware	2 depth cameras, IR emitter	2 cameras, 3 IR LEDs
Depth resolution	512 × 424 at 30 fps	640 × 240 at 60 fps
Tracking the user	Full body tracking	Hand tracking
Field of view	70° horizontal, 60° vertical	150° horizontal, 120° vertical
Measuring distance	50–450 cm ^1^	2.5–60 cm (−80 cm)
Measurement method	ToF	Mathematical methods
Access to raw data	Available	Available in recent versions

^1^ Measuring distance of the skeleton stream. The skeleton stream is compared to the LMC data as it is similar.
